# Regulation of *Emx2* Expression by Antisense Transcripts in Murine Cortico-Cerebral Precursors

**DOI:** 10.1371/journal.pone.0008658

**Published:** 2010-01-11

**Authors:** Giulia Spigoni, Chiara Gedressi, Antonello Mallamaci

**Affiliations:** International School for Advanced Studies (SISSA/ISAS), Trieste, Italy; INSERM, France

## Abstract

**Background:**

*Emx2* encodes for a transcription factor expressed in the embryonic intermediate mesoderm and central nervous system (CNS). It is implicated in several aspects of cerebral cortex development, including morphogenetic field specification, arealization, precursor proliferation and lamination. Four *Emx2*-associated antisense transcripts have been found in the urogenital system; one of them, *Emx2OS*, has been also detected in the adult brain. Until now, however, nothing is known about expression and function of *Emx2OS* in the developing CNS.

**Methodology/Principal Findings:**

By quantitative RT-PCR and in situ hybridization, we reconstructed the *Emx2OS* expression profile in the embryonic CNS, paying special attention to the developing cerebral cortex. *Emx2OS* was observed in a number of CNS structures expressing also *Emx2*. Within the cortex, *Emx2OS* was detectable in periventricular precursors, expressing the sense transcript, and peaked in newly born post-mitotic neurons not expressing such transcript. By integrating lentiviral gene delivery, RNAi, TetON technology, morpholino-mediated gene knock-down, drug-induced perturbation of gene expression, and quantitative RT-PCR, we addressed possible roles of *Ex2* antisense RNA in *Emx2* regulation, in primary CNS precursor cultures. We found that, in both cortical precursors and their neuronal progenies, *Emx2* antisense RNA contributes to post-transcriptional down-regulation of its sense partner, possibly by a Dicer-promoted mechanism. The same RNA, when delivered to rhombo-spinal precursors, stimulates ectopic expression of *Emx2*, whereas *Emx2* knock-out dramatically impairs *Emx2OS* transcription. This suggests that, within the developing CNS, a reciprocal *Emx2*/*Emx2OS* regulatory loop may normally sustain transcription at the *Emx2* locus.

**Conclusions/Significance:**

This study shows that antisense transcripts may contribute to developmental regulation of a key transcription factor gene implicated in CNS patterning, possibly by complex and multilevel mechanisms. The activation of *Emx2* by a short antisense transcript may be a prototype of a method for overexpressing *single specific* genes, without introducing additional copies of them into the genome.

## Introduction

*Emx2* is a transcription factor gene expressed in the developing urogenital and central nervous systems (CNS) [1], [3], crucial for proper morphogenesis of these structures [4], [6]. It is implicated in dorso-ventral specification of the rostral neural tube, conferring cortico-cerebral identity to precursors in the dorsal telencephalic vesicle and repressing the activation of striatal morphogenetic programs [7]. Expressed by cortical periventricular precursors along a caudal/medial^high^-to-rostral/lateral^low^ gradient, it further promotes the specification of hippocampus and occipital cortex, while antagonizing rostral-lateral areal programs [8]–[10]. Within the embryonic brain, it stimulates neural precursors' self-renewal, at expenses of neuronal differentiation; these effects are apparently reverted in post-natal neural stem cells [11]–[14]. Finally, *Emx2* is crucial to proper inside-out layering of the neocortical primordium. In its absence, pioneer Cajal-Retzius cells orchestrating such process are severely reduced and the neocortical lamination profile is deeply distorted, in a reeler-like way [15], [16].

Four main *Emx2* antisense transcripts of 6.0, 5.0, 2,3 and 1,8 kb have been detected in the murine urogenital system by Northern blotting and a cDNA corresponding to the second of them, *Emx2OS*, has been isolated. *Emx2OS* expression has been studied by *in situ* hybridisation in the uro-genital system: like *Emx2*-mRNA, *Emx2*OS-ncRNA is abundant in normal postmenopausal endometrium, reduced in premenopausal endometrium, absent/reduced in many primary endometrial tumors [17]. Moreover, its human counterpart has been specifically detected in the adult brain, by Northern blot [17]. However, the *Emx2OS* expression profile has not been studied in the developing CNS and little is known about possible implication of *Emx2*OS in fine regulation of *Emx2*.

Several transcription factor genes implicated in CNS development harbor antisense transcripts. Antisense transcripts are encoded by *Six3* [18], *Msx1* [19]–[23], *Pax6*, *Pax2*, *Six3*, *Six6*, *Otx2*, *Crx*, *Rax*, *Vax2* [24], *Dlx* 5/6 [25] and *Hox* loci [26]–[28]. More generally, antisense transcription is a genome-wide phenomenon, estimated to be associated with at least 10–40% of polypeptide-encoding genes [29]–[38]. Antisense/Sense (AS/S) pairs may be structurally classified as divergent, overlapping and convergent and it has been shown that they may display concordant, discordant or more complex expression patterns [39].

Based on various relationships occurring among AS expression levels and those of their sense partners, it has been predicted that the former ones might differentially promote or antagonize the expression of such partners, depending on the gene and on the context. Such prediction has been substantially confirmed in a variety of functional studies, indicating that: (1) AS transcription is not simple transcriptional noise or epiphenomenon of S transcription [36], but often results a prerequisite for proper regulation of “coding” sense genes; (2) molecular mechanisms by which AS transcripts act are extremely complex and largely diversified [39], [40].

In some cases, poor sequence conservation but similar exon/intron organisation characterizing antisense transcripts from different species suggests that transcription *per se*, and not the AS RNA molecule, may be crucial to the function. That has been demonstrated in the case of the silent human provirus HERV-K18, where AS transcription promotes S transcription [41], as well as in a variety of other cases, where AS transcription conversely inhibits S transcription, because of competition between the two transcriptional machineries for shared cofactors, or due to collision between them [42].

However, the AS transcript may also work as such, acting at distinctive regulatory levels. First, it may regulate the epigenetic state of chromatine, modulating the methylation state of DNA and/or histones [27], [43], [44]. Second, it may facilitate recruitment of transcription factors to enhancers impinging on the partner sense gene, so promoting its transcription. This is the case of the *Evf2* ncRNA, cooperating with the homeoprotein Dlx2 in stimulating transcription of its sense partner *Dlx6* [25]. Third, the AS ncRNA may modulate the splicing of its partner sense pre-mRNA, as reported for the *TRa2* and *Zeb2* loci [45], [46]. Fourth, AS transcripts may regulate the half-life of their sense partners. Pairing of retrotransposon sense and antisense transcripts paves the way to Dicer-dependent cutting of resulting dsRNAs, followed by siRNA-instructed silencing of sense transcripts, from the same or paralogous loci [47]–[49]. Fifth, antisense RNAs may modulate translation [50]. Last, they may be necessary for proper activity of their sense partners, as shown for the *Xist/Tsix* pair [51].

In this study, we investigated the expression pattern of *Emx2OS*-ncRNA in the developing CNS, by quantitative reverse transcription polymerase chain reaction (qRT-PCR) and *in situ* hybridisation (ISH). Then, by integrating lentiviral cDNA delivery, drug-induced perturbation of patterning pathways and gain- or loss-of-function (GOF and LOF, respectively) designs, we addressed possible roles of this transcript on regulation of its partner gene, *Emx2*, on primary CNS cultures as well as on cell lines. We found that *Emx2* antisense transcripts may both stimulate and refine *Emx2* expression. Moreover, such effects seem to be exerted at two distinct regulatory levels, transcriptional and post-transcriptional.

These results, beyond their contribution to the comprehension of mechanisms regulating cortico-cerebral development, suggest a possible general exploitation of AS-based methods, as a tool for artificial triggering of endogenous gene expression.

## Results

### Expression Pattern of Emx2OS-ncRNA

**Figure 1:**
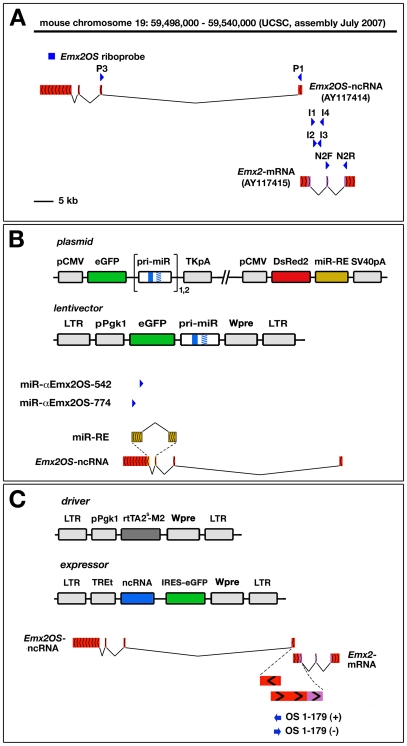
Synopsis of molecular tools used for studying expression and function of *Emx2OS*. (A) Representation of the murine *Emx2* locus, with the *Emx2*-mRNA and the *Emx2OS*-ncRNA transcription units (red). Genomic localization of the riboprobe used for *in situ* hybridization analysis of *Emx2OS* (blue bar). Genomic localization of the oligonucleotides employed for quantitative RT-PCR evaluation of *Emx2OS*-ncRNA (P1 and P3), *Emx2*-mRNA (N2F and N2R) and *Emx2*-pre-mRNA (I1, I2, I3 and I4) (blue arrowheads). (B) Structure of the bi-cistronic plasmids employed for assaying activities of artificial miRNAs against *Emx2OS*-ncRNA in HeLa cells and of lentivectors for overexpressing these miRNAs in primary cells. Localization of miR-α*Emx2*OS-542 and -774 (blue arrowheads) as well as of their responsive element, *miR-RE*, with respect to the antisense transcript. *miR-RE* (dark yellow), showed enlarged over the antisense transcript, extends across the 3^rd^ and the 4^th^ exons of it. (C) Structure of the “driver” lentivector, guiding constitutive expression of rtTA2^S^-M2, and of “expressor” lentivectors, guiding rtTA/doxycycline-dependent expression of ncRNA/IRES/eGFPcds modules. Genomic localization of OS1-179(+) and OS1-179(−) ncRNAs (blue arrows), as compared to *Emx2OS*-ncRNA and *Emx2*-mRNA (for sake of clarity, the sense/antisense ovarlapping region and its surroundings are represented enlarged; red, non coding sequences; violet, coding sequences).

**Figure 2:**
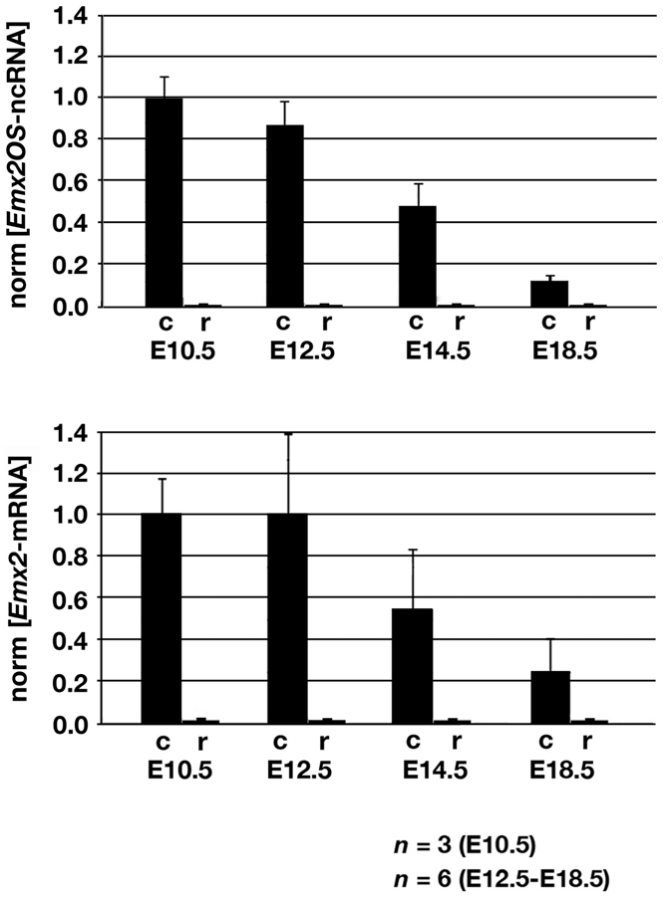
Time-course quantitative RT-PCR analysis of *Emx2*-mRNA and *Emx2OS*-ncRNA expression in the developing cortex and rhombencephalon. Sense and antisense transcripts display concordant spatial distributions (abundant in the cortex, absent in rhombencephalon), from E10.5 to E18.5. Within the cortex, they share a similar temporal trend, being progressively down-regulated from the pre-neuronogenic (E10.5) to post-neuronogenic stages (E18.5). RTs are primed by random hexamers and PCRs by oligos shown in Fig. 1. Data are normalized on E10.5 cortical samples. Abbreviations: c, cortex; r, rhombencephalon.

To get a first insight into expression and putative functions of *Emx2OS*-ncRNA, we compared its abundance with that of *Emx2*-mRNA, in cortico-cerebral and rhomboencephalic tissues, at four embryonic developmental ages, E10.5, E12.5, E14.5 and E18.5. As expected, *Emx2*-mRNA was specifically detectable in the cortex, where its levels went progressively down from E10.5 up to E18.5 [3], [52]. Interestingly, *Emx2OS*-ncRNA displayed similar spatial specificity and similar temporal progression (Fig. 1A and 2).

**Figure 3:**
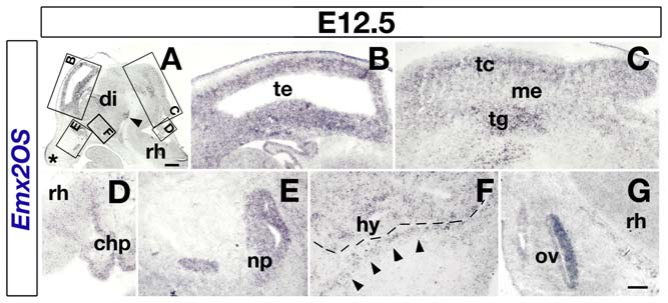
Distribution of *Emx2OS* transcripts in the embryonic E12.5 mouse brain. (A–D) Within the E12.5 developing CNS, *Emx2OS*-ncRNA is detectable in telencephalon (A,B), mesencephalon (me), including both tectum (te) and tegmentum (tg), (panels A,C) and diencephalon, including the mammillary recess (arrowhead in panel A). It is not present in rhombencephalon (rh, panel G), except the choroid plexus of the IV ventricle (chp, panels A,D). Outside the CNS, *Emx2OS* is expressed in primordia of sense organs, nasal pits (np, panel E) and otic vesicle (ov, panel G), as well as in mesenchyme underlying snout epidermis (A, asterisk) and surrounding the hypothalamic (hy) optic recess (F, arrowheads). Scalebars, 200 µm in A–C, 50 µm in D–G.

**Figure 4:**
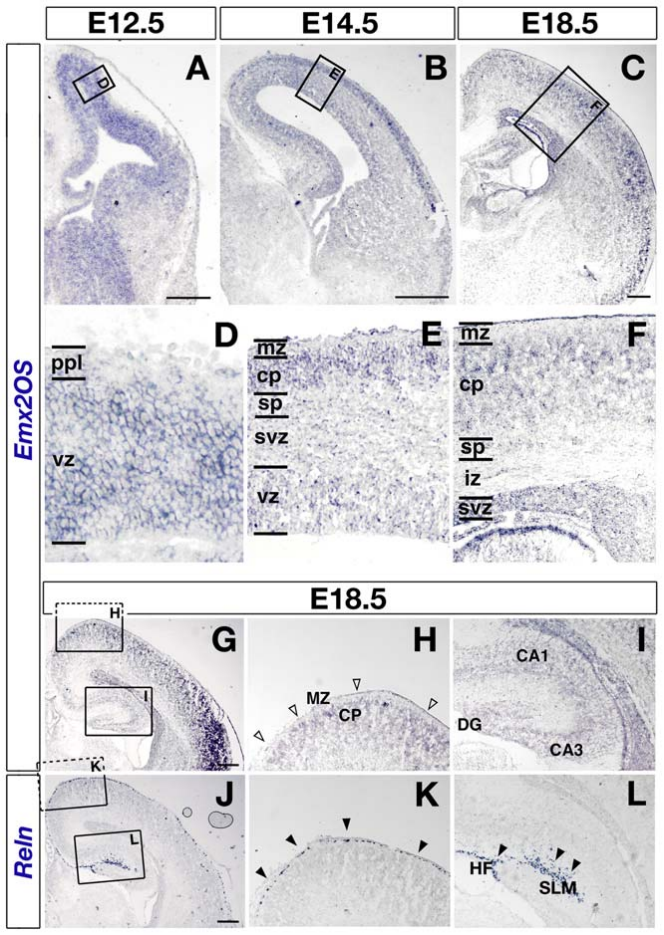
*In situ* hybridization profile of Emx2OS-ncRNA in the developing mouse telencephalon. Distribution of *Emx2OS*-ncRNA (A–I) and *Reln*-mRNA (J–L) on mid-frontal sections of E12.5 (A), E14.5 (B) and E18.5 (C,G,J) mouse telencephalons. (D), (E) and (F) are magnifications of boxed areas in (A), (B) and (C), respectively. (H) and (I) are enlargements of boxed areas of panel (G), respectively; the same applies to panels (K) and (L) with respect to (J); (G) and (J) are adjacent sections from the same brain. *Emx2OS* is expressed in periventricular proliferative layers at all ages subject of analysis (A–C). An additional signal is detectable within the cortical plate starting from E14.5 (B) and gets confined to its marginal-most part at E18.5 (C). No *Emx2OS* expression can be detected in the neocortical marginal zone (G,H, empty arrowheads) as well as in the archicortical stratum lacunosum-moleculare (G,I), both rich of *Reln* ^+^ Cajal-Retzius cells (J–L, solid arrowheads). Some aspecific staining may be found in meninges, at the edge of sections. Abbreviations: CA1, cornu Ammonis field 1; CA3, cornu Ammonis field 3; cp, CP, cortical plate; DG, dentate gyrus; HF, hippocampal fissure; iz, intermediate zone; mz, MZ, marginal zone; ppl, preplate; SLM, stratum lacunosum-moleculare; sp, subplate; svz, subventricular zone; vz, ventricular zone; Scalebars, 200 µm.

Then, we studied the *Emx2OS*-ncRNA expression pattern in the developing central nervous system (CNS), by non-radioactive *in situ* hybridisation (Fig. 1A). At E12.5, within the CNS, the transcript was detectable in the telencephalon (Fig. 3A,B), including both pallium and basal ganglia (Fig. 4A), in the mammillary recess of the hypothalamus (Fig. 3A, arrowhead) and in the midbrain, including tectum and tegmentum (Fig. 3C). No signal was detectable within the rhombo-spinal domain (Fig. 3A,D). *Emx2OS* was also expressed by the nasal pits (Fig. 3E), the otic vesicle (Fig. 3G), the choroid plexus of II and IV ventricle (Fig. 3A,D), as well as by two clusters of head mesenchyme cells, in the snout region (Fig. 3A, asterisk) and in the surroundings of the hypothalamic optic recess (Fig. 3F, arrowheads).

Focussing our attention on the developing cerebral cortex, we found *Emx2OS* transcripts within periventricular proliferative layers, from E12.5 to E18.5 (Fig. 4A–F), as well as in the cortical plate (CP), especially in its more superficial part (Fig. 4E,F). Conversely, no signal was detectable within the preplate (PPL) and its derivatives, marginal zone (MZ) and subplate (SP) (Fig. 4D–F). In particular, no *Emx2OS* transcripts could be found within Cajal-Retzius (CR) cells, aligned beneath the *pia mater* and specifically expressing *Reln* mRNA, in both neocortex and hippocampus (Fig. 4G–L, empty and solid arrowheads).

### Emx2OS Antagonizes Emx2 Expression

The mutually exclusive distribution of *Emx2*-mRNA and *Emx2*OS-ncRNA among cortico-cerebral neurons (the former expressed by CR neurons, the latter by CP neurons) suggested to us that reciprocal down-regulation between them might occur and that, in particular, *Emx2*OS-ncRNA might be implicated in repression of *Emx2*mRNA.

**Figure 5:**
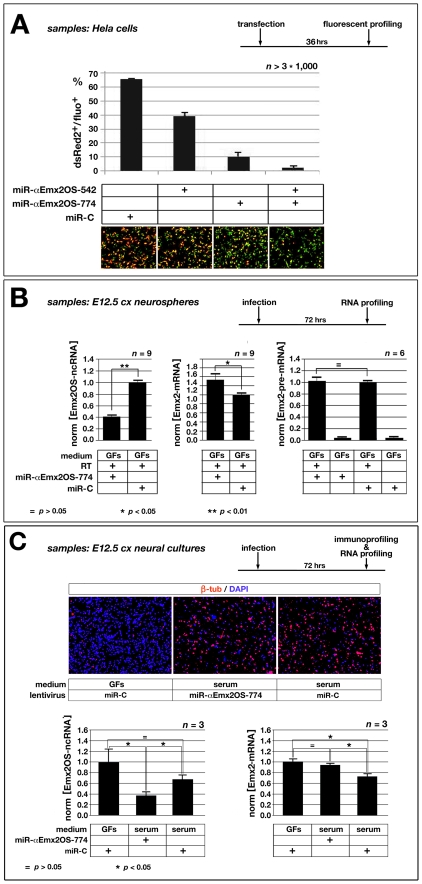
*Emx2OS* knock-down in HeLa cells and dissociated cortico-cerebral precursors. (A) Down-regulation of the miR-RE-sensitized *DsRed2* reporter in Hela cells, upon overexpression of artificial miRNAs designed against *Emx2OS*. (B) Down-regulation of *Emx2OS*-ncRNA, up-regulation of mature *Emx2*-mRNA and unchanged levels of immature *Emx2*-pre-mRNA in E12.5-derived cortical primary cells (cx), acutely infected by miR-αEmx2OS-774-expressor lentivirus, kept in Sato medium and harvested 72 hours later. Data are normalized on control-miR-treated samples (miR-C). (C) Neuronal differentiation of lentivirus-transduced neural precursors kept 72 hours under 5% serum, as assessed by β-tubulin immunoprofiling. Down-regulation of *Emx2*-mRNA in miR-C-infected neural precursors, kept 72 hours under 5% serum in place of growth factors (GFs). Rescue of such down-regulation, elicited via miR-αEmx2OS-774-induced knock-down of *Emx2OS*-ncRNA. qRT-PCR data are normalized on control-miR-treated samples (miR-C), kept under GFs.

We first assayed this hypothesis by a LOF approach, i.e., by knocking-down *Emx2*OS-ncRNA in primary neurospheres derived from E12.5 cortical tissue, via RNAi, and monitoring consequences of that on *Emx2*-mRNA levels. For this purpose, we used a lentivirus constitutively expressing miR-α*Emx2*OS-774 (Fig. 1B), an artificial miR able to target Emx2OS sequences in HeLa cells (Fig. 5A). Under miR-α*Emx2*OS-774, *Emx2*OS-ncRNA signal was reduced by more than 2 times (p<0.002; n = 9) (Fig. 5B, left). Concomitantly, we observed a modest (+25%), but statistically significant (p<0.03; n = 9) increase of *Emx2*-mRNA.

To assess if upregulation of *Emx2* induced by miR-α*Emx2*OS-774 took place at transcriptional or post-transcriptional level, we then measured *Emx2* pre-mRNA levels, following infection of neurospheres with miR-α*Emx2*OS-774 or negative control lentiviruses. For sake of sensitivity and specificity, measurements were done by two-step quantitative RT-PCR, using two sets of nested primers annealing within the first *Emx2* intron (Fig. 1A). Interestingly, no change in *Emx2* pre-mRNA levels was found (Fig. 5B, right), suggesting that *Emx2OS*-dependent regulation of *Emx2* took place at post-transcriptional level.

Remarkably, in previous sets of experiments, neural cultures were performed in DMEM/F12/N2 medium, containing the standard growth factors (GFs) mix which promotes the intermitotic/stem state [53]. As during embryonic development *Emx2*-mRNA disappears in post-mitotic neurons, where Emx2OS-ncRNA is transiently upregulated, the question arises: is *Emx2OS*-ncRNA able to down-regulate *Emx2*-mRNA *also* in nascent neurons? To address this issue, the *Emx2*OS-LOF tests were repeated culturing the infected cells in the presence of 5% serum, which stimulates neuronal differentiation, in place of GFs. Moreover, in this new experiment an aliquot of miR-C-infected cells was kept under GFs, as a control. Consistently with our expectations, under 5% serum plus miR-C lentivirus, neuronal differentiation was dramatically stimulated, as confirmed by massive activation of neuron-specific β-tubulin (Fig. 5C, top), *Emx2*-mRNA level decreased, by about 30% (Fig. 5C, bottom-right), and *Emx2*OS-ncRNA was only slightly downregulated (Fig. 5C, bottom-left). Remarkably, knock-down of *Emx2*OS induced by miR-α*Emx2*OS-774 rescued the *Emx2*-mRNA decrease to large extent (Fig. 5C, bottom-right), suggesting that *Emx2*OS-ncRNA substantially contributes to *Emx2* down-regulation at the time when CP neurons are born.

Then, to confirm this model, we verified it by a complementary GOF approach, i.e. by delivering antisense *Emx2* cDNA to embryo-derived neurospheres, via lentiviral vectors, and scoring consequences of that.

Unfortunately, the full-length 5.0 kb *Emx2*OS cDNA harbors several canonical polyadenylation signals, which make it hardly suitable for our lentiviral expression system. So, in place of it, we overexpressed its 5′-most 179 bp fragment, coinciding with the *Emx2-mRNA*/*Emx2OS*-ncRNA overlapping region minus a low-complexity 74 bp polypyrimidine tract. Assuming - in fact - that *Emx2*OS-ncRNA works by interacting with *Emx2*-mRNA via Watson&Crick base-pairing, this short fragment could recapitulate key regulative properties of the full-length *Emx2OS*-ncRNA. Moreover, in order to assay both consequences of antisense overexpression and their reversibility, we performed *Emx2OS*-GOF manipulations by a dual, TetON-based, lentiviral delivery system, triggered by doxycycline. Neurospheres were infected by a “driver virus”, harboring a constitutively expressed rtTA2^S^-M2 transactivator gene, plus an “expressor virus”, harboring the cDNA sequence subject of investigation (Fig. 1C).

**Figure 6:**
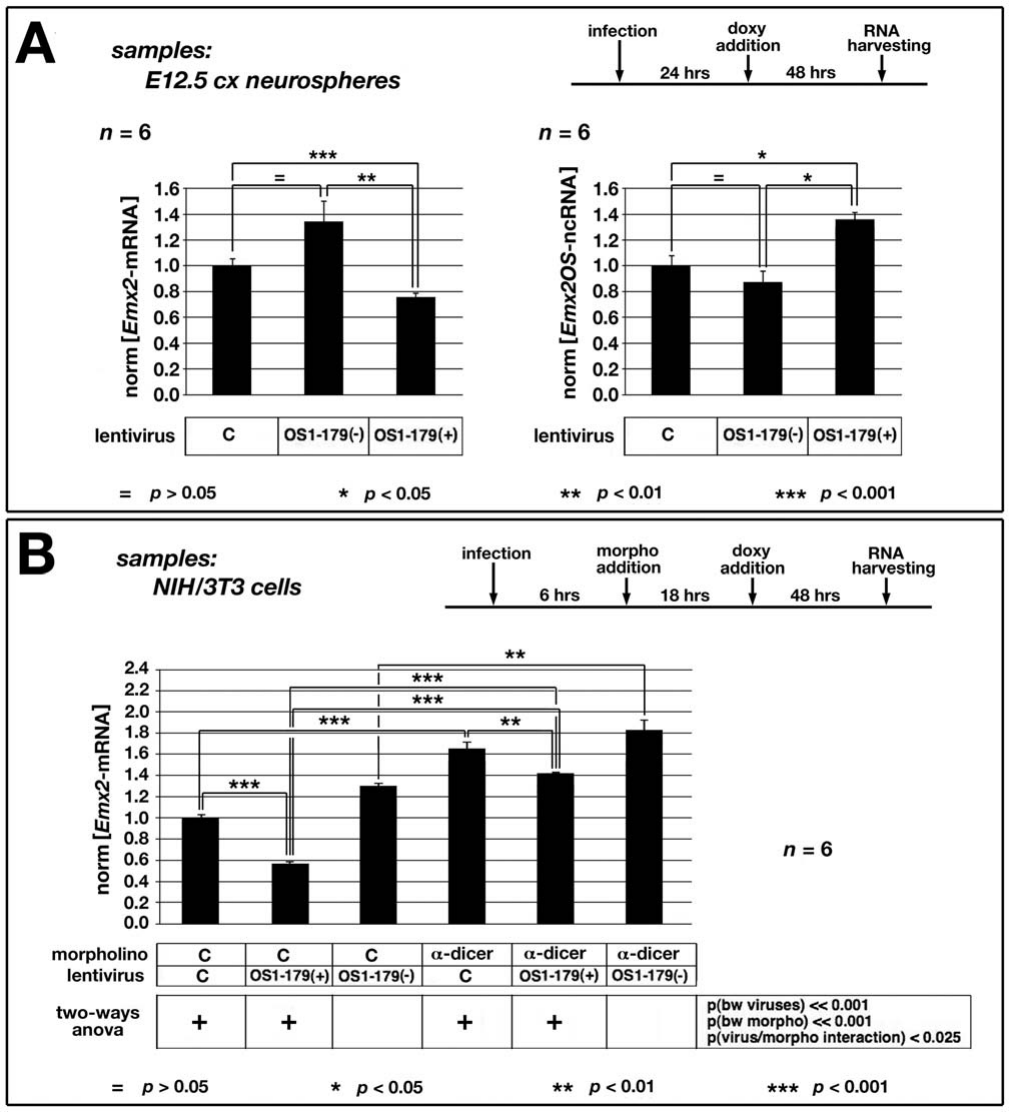
*Emx2* and *Emx2OS* expression in dissociated cortico-cerebral precursors and NIH/3T3 cells upon OS1-179(+) delivery. (A) *Emx2*-mRNA and *Emx2OS*-ncRNA expression in primary neural precursor cells, derived from E12.5 cortices (cx) and acutely infected with the “driver” lentivector, plus “expressor” lentivectors [OS1-179(+), OS1-179(–) and control (C)] in different combinations. One day after sample dissociation, cells are administered with doxycycline and, two more days later, profiled for RNA. Data are normalized on control lentivirus-treated samples. OS1-179(+) induces a moderate, but statistically significant down-regulation of *Emx2*-mRNA, as well as a moderate up-regulation of the endogenous antisense transcript. (B) *Emx2*-mRNA expression in NIH/3T3 cells, acutely infected with the “driver” lentivector plus each of the three “expressor” lentivectors [OS1-179(+), OS1-179(-) or C]. Cells are administered 6 hours after infection with an anti-Dicer1 or a control (C) morpholino and 18 more hours later with doxycycline. Two more days later, they are RNA-profiled by qRT-PCR. Data reported in the histogram are normalized on samples infected by control lentivirus and exposed to control morpholino. Compared with lentivirus-C-treated controls, levels of *Emx2-mRNA* change upon OS1-179(+) infection by a factor of 0.56/1 = 0.56 (p<0.001) and 1.42/1.65 = 0.87 (p<0.01), in NIH/3T3 cells treated by control morpholino and α-Dicer1, respectively. Compared with morpholino-C-treated controls, levels of *Emx2*-mRNA change upon α-Dicer1 administration by a factor of 1.42/0.56 = 2.54 (p<0.001), 1.65/1 = 1.65 (p<0.001), and 1.82/1.31 = 1.39 (p<0.01), in cells infected by OS1-179(+), control or OS1-179(–) lentiviruses, respectively. Two-ways ANOVA data analysis suggests the occurrence of a functional interaction between Dicer1 and OS1-179(+), with p<0.025 (Fig. 6B).

Compared to the control, the *Emx2* antisense-encoding lentivirus OS1-179(+) reduced the *Emx2*-mRNA level by 24.2% (p<0.001). OS1-179(−), encoding for the corresponding reverse-complementary sequence, increased it by 34.2% (while not reaching statistical significance). Moreover, *Emx2-mRNA* levels under OS1-179(+) and OS1-179(−) differred by −43.7% (p<0.01). Remarkably, these changes of *Emx2*-mRNA levels were mirrored by opposite concomitant changes of *Emx2OS*-ncRNA (Fig. 6A). In synthesis, OS1-179(+) both down-regulates *Emx2*-mRNA and promotes the expression of the endogenous antisense transcript, which may in turn contribute to *Emx2* down-regulation.

As for molecular mechanisms mediating *Emx2*OS-dependent *Emx2-mRNA* down-regulation, we hypothesized that the antisense ncRNA might destabilize the sense mRNA, forming a double strand with it and so preparing its degradation by double-strand ribonucleases. Among these enzymes, a reasonable candidate might be Dicer1, involved in dsRNA-mediated destabilization of retrotransposon transcripts [47]-[49] and crucial to generation and survival of cortico-cerebral neurons [54]. To test this hypothesis, we decided to repeat the GOF experiments described above in cells alternatively provided with Dicer1 activity or deprived of it, by morpholino technology [55], [56]. To get the best Dicer1 knock-down, NIH/3T3 cells, among the easiest to transfect by established morpholino reagents, were selected as a substrate.

Combined Dicer1-knock-down (DicerKD) by α-Dicer1 morpholino and OS1-179(+)/OS1-179(-)-GOF manipulations were performed, according to the schedule in Fig. 6B. Results were as follows. Lentivirus OS1-179(+) downregulated *Emx2-*mRNA in all tested conditions. Compared with lentivirus-C treated controls, relative levels of *Emx2-mRNA* upon OS1-179(+) infection, changed by a factor of 0.56/1 = 0.56 (p<0.001) - and 1.42/1.65 = 0.87 (p<0.01), in wild type and Dicer1-KD NIH/3T3 cells, respectively. Morpholino α-Dicer1 gave conversely rise to a robust increase of *Emx2*-mRNA. Compared with morpholino-C-treated controls, relative levels of *Emx2*-mRNA under α-Dicer1 changed by a factor of 1.42/0.56 = 2.54 (p<0.001), 1.65/1 = 1.65 (p<0.001), and 1.82/1.31 = 1.39 (p<0.01), in cells infected by OS1-179(+), control or OS1-179(−) lentiviruses, respectively.

The stronger effect elicited by OS1-179(+) in the presence of normal levels of Dicer1 and the more pronounced effect elicited by α-Dicer1 when associated to OS1-179(+) suggested that a specific functional interaction could occur between Dicer1 and the antisense transcript. Remarkably, two-ways ANOVA re-analysis of *Emx2* expression data confirmed this suspect, with p<0.025 (Fig. 6B).

Summarizing: (1) *Emx2-mRNA* is normally down-regulated by Dicer1; (2) the antisense RNA fragment overlapping *Emx2*-mRNA destabilizes it; (3) Dicer1 promotes such antisense-dependent mRNA destabilization.

### Emx2OS Transcripts Promote Both Emx2-mRNA and Emx2OS-ncRNA Expression

**Figure 7:**
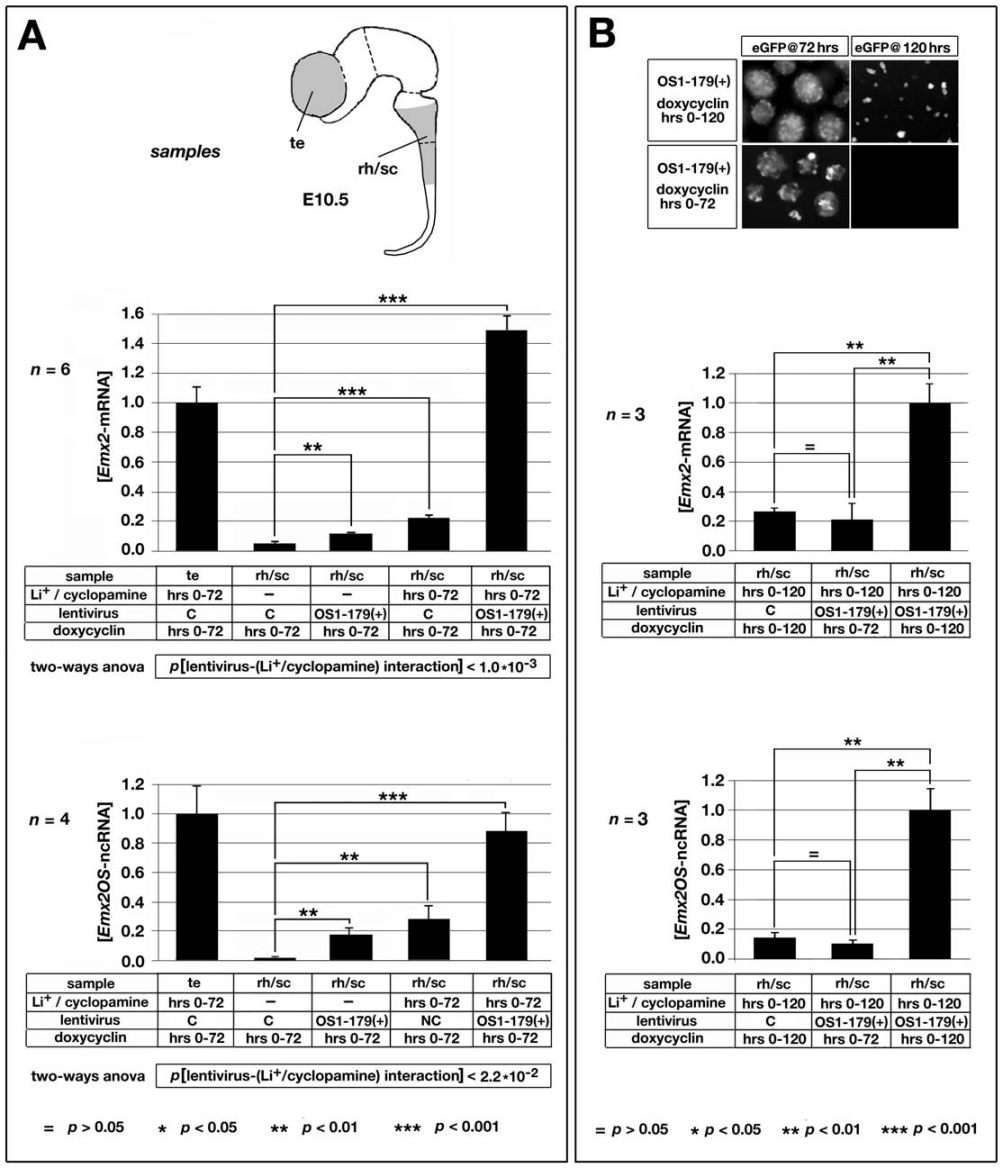
Ectopic activation of *Emx2*-mRNA and *Emx2OS*-ncRNA in rhombo-spinal neurospheres, upon OS1-179(+) delivery. (A) Ectopic activation of the endogenous *Emx2* locus by OS1-179(+). The two graphs show *Emx2*-mRNA and *Emx2OS*-ncRNA expression levels in primary neural precursors from distinctive portions of the E10.5 CNS, rhombo-spinal tract (rh/sc) and telencephalon (te), acutely infected with “driver” and “expressor” lentivectors (Fig. 1C), kept under doxycycline and added or not with the Li^+^/cyclopamine mix. Samples are RNA-profiled 72 hours after infection. Data are normalized on telencephalic precursors exposed to Li^+^/cyclopamine, infected by control lentivirus and kept under doxycycline. Basal *Emx2* expression by rhombo-spinal cells, very low as compared to telencephalic precursors, rises about 2 (p<0.01), 4 (p<0.001) and 30 times (p<0.001), upon treatment of these cells with the OS1-179(+) virus, the drug mix, or both, respectively. Two-ways ANOVA indicates that a specific interaction between OS1-179(+) and the drug mix takes place, with p<0.001. A similar course is shown by *Emx2OS*-ncRNA. (B) Reversibility of OS1-179(+)-dependent activation of the endogenous *Emx2* transcription unit. The four panels to the top show time course analysis of eGFP fluorescence in neural precursors, infected by driver and OS1-179(+) lentiviruses and kept under doxycycline for 72 or 120 hours. The absence of fluorescence in the bottom-right panel means that withdrawal of doxycycline at 72 hours is sufficient to reset levels of the eGFP/OS1-179(+) chimaeric transcript to zero by 120 hours. The two graphs to the bottom show *Emx2*-mRNA and *Emx2OS*-ncRNA expression levels in primary neural precursors from the E10.5 rhombo-spinal (rh/sc) tract, acutely infected with “driver” and “expressor” lentivectors (Fig. 1C), kept under doxycycline for 72 or 120 hours and chronically exposed to Li^+^/cyclopamine throughout the experiment. Samples are RNA-profiled 120 hours after infection. Data are normalized on rhombo-spinal cells exposed to Li^+^/cyclopamine, infected by OS1-179(+) lentivirus and kept under doxycycline throughout the experiment. Removal of doxycycline at 72 hours abolishes (p<0.01) the 4-fold up-regulation of *Emx2*-mRNA, detectable in Li/cyclopamine-treated rhombo-spinal precursors, upon their further infection by lentivirus OS1-179(+) (p<0.01). Similar consequences are elicited by doxycycline removal on levels of *Emx2OS*-ncRNA.

The specific co-expression of *Emx2OS*-ncRNA and *Emx2-*mRNA by neural precursors belonging to defined regions of the developing CNS suggested that antisense transcripts, in addition to trigger destabilization of *Emx2*-mRNA, might be also basically implicated in promoting its transcription. This hypothesis was tested by over-expressing *Emx2* antisense sequences in neural precursors from the rhombo-spinal tract, a CNS segment which normally does not express *Emx2* [1], [2] (Fig. 1C). As basal expression levels of *Emx2* in rhombo-spinal precursors are very low (near the experimental background) and this might make it difficult to estimate the relative change of transcript level elicited by OS1-179(+), the following device was adopted. The experiment was alternatively run under standard neurosphere medium [15], or upon addition of a specific lithium/cyclopamine drug mix, which, stabilizing the transcription factor (TF) beta-catenin [57] and preventing inhibition of the TF Gli3 [58], promotes *Emx2* transcription [59]–[61], so as to increase the baseline of the assay and ameliorate its signal-to-noise ratio (Fig. 7A).

As expected, the *Emx2* expression level in rhombo-spinal cells, infected with control virus and kept in the absence of drugs, resulted far lower than in telencephalic cells exposed to lithium/cyclopamine. The *Emx2* level slightly rose, upon treatment of rhombo-spinal cells by either the OS1-179(+) virus or the lithium/cyclopamine mix (about 2 and 4 times, with p<0.01 and p<0.001, respectively). Interestingly, simultaneous exposure of rhombo-spinal cells to both OS1-179(+) virus and lithium/cyclopamine elicited a much more dramatic up-regulation effect (almost 30 times, with p≪0.001), so confirming the effectiveness of the OS1-179(+) virus and further suggesting a powerful synergy between such virus and the drug cocktail (p<0.001) (Fig. 7A, upper histograms). In other words, as hypothesized, *Emx2OS* transcripts may activate *Emx2* expression and the amplitude of this phenomenon is much more prominent, upon appropriate pharmacological modulation of the nuclear TF milieu impinging on *Emx2* regulation [57], [59]. Noticeably, changes of *Emx2*-mRNA levels elicited by pharmacological and genetic manipulations described above were paralleled by strikingly similar changes of *Emx2OS*-ncRNA (Fig. 7A, lower histograms). This indicates that both reasonably originated from concerted stimulation of sense and antisense transcription at the *Emx2* locus. This also suggests that the upregulation of *Emx2*-mRNA triggered by artificial OS1-179(+) administration might be further sustained by endogenous antisense transcripts from the same locus, induced by OS1-179(+) itself (Fig. 7A).

As the previous experiment was done by keeping the OS1-179(+) transgene chronically on, it was poorly informative about the kinetics and, in particular, the reversibility of the *Emx2* activation triggered by antisense transcripts. To address this issue, we repeated the OS1-179(+) overexpression test over 120 hours, limiting neural precursors exposure to doxycycline to the first 72 hours (Fig. 7B).

Results of this test were as follows. Withdrawal of doxycycline at 72 hours allowed full shutting off of OS1-179(+)-ncRNA at 120 hours, as witnessed by the absence of associated eGFP fluorescence (Fig. 7B, panels to the top). Remarkably, this was accompanied by a collapse of endogenous *Emx2* sense and antisense transcripts to levels peculiar to rhombo-spinal cells infected by the control virus (Fig. 7B, histograms).

In other words, even low, but *persisting* levels of antisense transcripts seem to be *sufficient* and strictly *necessary* for proper firing of the *Emx2* transcription unit. This might explain why *Emx2* sense and antisense transcripts have normally to be co-expressed in proliferative layers of the early developing CNS.

### Absence of Emx2 Sense Transcription and/or Its Products Impairs Emx2OS-ncRNA Expression

The association between *Emx2OS* and *Emx2* transcripts might also imply a reciprocal, complementary dependence of *Emx2OS*-ncRNA expression on *Emx2* sense transcription. To address this issue, we compared levels of *Emx2OS* transcripts in E12.5 cortices from *Emx2^+/+^*, *Emx2^+/−^* and *Emx2^−/−^* mouse embryos [4]. The *Emx2 null* allele of these mutants - in fact - lacks only a 250bp genomic fragment encoding for the C-term of the homeodomain (not provided of any known *cis*-regulatory activities), has an intact divergent-promoter region and still harbors the two main enhancers which drive transcription in the developing telencephalon [4], [57]. As such, it should in principle be able to normally drive the synthesis of its antisense transcript.

**Figure 8:**
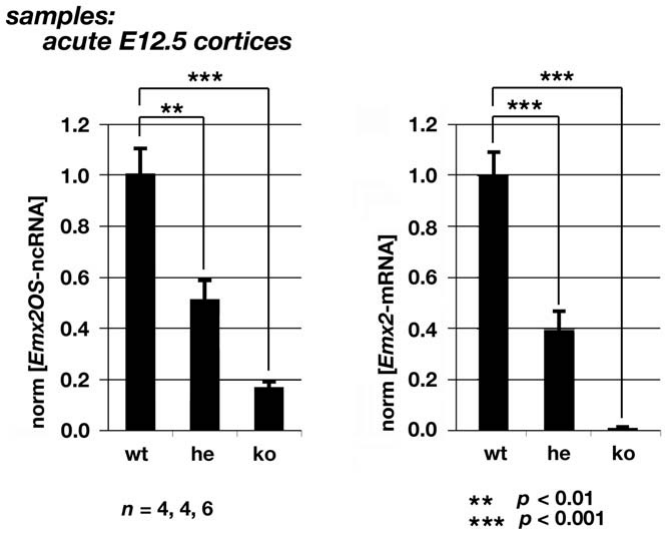
Down-regulation of *Emx2*-mRNA and *Emx2OS*-ncRNA in acutely dissected cortices from E12.5 *Emx2* null mutant embryos. Abbreviations: wt, wild type; he, heterozygous; ko, knock-out.

Notwithstanding that, we found that *Emx2OS*-ncRNA was down-regulated by more than 80% in homozygous null mutants and by 50% in heterozygous mutants (Fig. 8), suggesting that - as suspected - *Emx2* sense transcription and/or its products are in turn necessary for proper expression of *Emx2OS*-ncRNA.

## Discussion

In this study we investigated the expression pattern of *Emx2OS*-ncRNA, the antisense transcript associated to the transcription factor gene *Emx2*, in the developing mouse CNS. Then, we preliminarly addressed its involvement in regulation of *Emx2* expression.

As shown by qRT-PCR (Fig. 2) and *in situ* hybridisation (Fig. 3), *Emx2OS* is specifically expressed by a number of CNS substructures and sense organs also expressing *Emx2*. Among them: telencephalon, mammillary recess, optic tectum, olfactory placode, otic vesicle [1], [3]. Such colocalization of the two transcripts in periventricular layers of defined neural tube domains recalls the identical expression patterns they display in the uterine endometrium [17]. *Emx2*/*Emx2*OS colocalization might be an epiphenomenon of shared regulatory mechanisms impinging on both. Alternatively, transcription of one or both units might specifically promote the other and, in particular, antisense transcription might contribute to specifically keep *Emx2* on in neural precursors of defined spatial domains.

Remarkably, the two transcripts, both expressed by periventricular neural precursors of the cortical primordium, display a mutually exclusive pattern in post-mitotic progenies of such precursors. Newborn neurons belonging to the cortical plate strongly express *Emx2OS*-ncRNA, expecially at the end of their radial migration (Fig. 4E,F), but not *Emx2*-mRNA (while still harboring residual Emx2 immunoreactivity) [1]-[3]. Pioneer Cajal-Retzius neurons lying in the marginal zone conversely express huge amounts of *Emx2* mRNA and protein [3], but no antisense transcript at all (Fig. 4G,L). Such mutual distribution recalls the complementary expression patterns displayed by *Otx2*/*Otx2*OS and *Crx*/*Crx*OS pairs in the adult retina, with the sense transcription factor genes expressed predominantly in photoreceptors and the corresponding OS genes in bipolar interneurons and ganglionic projection neurons [24]. The mutual distribution of *Emx2* and *Emx2*OS RNA products points to a possible negative cross-regulation between them and, in particular, to an involvement of the former in fine control of post-mitotic silencing of the latter.

As for the biological meaning of *Emx2*/*Emx2OS* co-expression, we tested the hypothesis that sense and antisense transcription from the *Emx2* locus might reciprocally sustain each other.

To assay if *Emx2OS*-ncRNA is able to promote *Emx2*-mRNA expression, a 5′ fragment of the former was overexpressed in rhombo-spinal precursors, normally expressing none of them. As suspected, delivery of this fragment, OS1-179(+)-ncRNA, into such cells elicits strong *Emx2* upregulation. No direct assessment of the level, transcriptional or post-transcriptional, at which this phenomenon takes place, was performed. However two considerations suggests that the former possibility may hold good. First, OS1-179(+) synergizes with a drug mix specifically up-regulating beta-catenin, a key trans-activator binding the two telencephalic enhancers of *Emx2* [59]. Second, up-regulation of *Emx2* is faithfully paralleled by *Emx2OS* up-regulation.

Concerning mechanisms mediating transactivating properties of OS1-179(+) (as well of *Emx2OS*-ncRNA), two hypotheses may be taken into accont. Both molecules might interact with transcription factors impinging on the *Emx2* locus, ameliorating their binding to the chromatin and/or their processivity (like the *Evf2*-ncRNA at the *Dlx5*/6 locus) [25]. Alternatively, antisense transcripts or their by-products might make the epigenetic state of *Emx2* chromatine more permissive to gene expression, by recruiting appropriate modifier enzymes to it. However, modifier enzymes recruited by antisense RNAs, rather than facilitating gene expression, often make chromatine less prone to transcription [27], [43], [44]. Consistent with these considerations, the need of *persistent* OS1-179(+) expression to get *Emx2* activation and the capability of the only drug cocktail to elicit weak but reproducible *Emx2* upregulation induce us to rule out the latter hypothesis and to consider the former more likely. Beyond mechanics of OS1-179(+) action, such activation of *Emx2* by a short antisense transcript is nonetheless remarkable, as possible prototype of a general method for overexpressing *single specific* genes, without any need to introduce additional copies of them into the genome, similarly to classical RNAa [62]–[65].

To ascertain if *Emx2OS*-ncRNA expression reciprocally depends on *Emx2* transcription (and/or its products), *Emx2OS* levels were scored in *Emx2* mutant mice, not harboring any gene lesions obviously incompatible with *Emx2OS* transcription. Remarkably, in such mice, both *Emx2* and *Emx2OS* transcripts are dramatically down-regulated, echoing the collapse of *Vax2*OS (*Vax2* opposite strand) occurring in mice knock-out for the cognate transcription factor gene *Vax2* [24]. Multiple are mechanisms possibly underlying such down-regulation. Among the most likely ones, the depression of the *Emx2*/*Wnt* mutually-sustaining loop [9], [59], leading to a decrease of the nuclear beta-catenin pool which normally stimulates transcription at the *Emx2* locus [59]. Such mechanisms - however - were not addressed at all in this study; they will be subject of a further dedicated one.

So, based on *Emx2OS* down-regulation in *Emx2*-null mutants as well as on consequences of OS1-179(+) overexpression in rhombo-spinal precursors, we can speculate now that *mutual* promotion of sense and antisense transcription at the *Emx2* locus may be a crucial pre-requisite for proper activation and expression of *Emx2*. Maybe that is why *Emx2* sense and antisense transcripts have to be co-expressed in defined domains of the early neural tube.

We also tested whether *Emx2*OS is also implicated in downregulation of *Emx2*, by artificially modulating *Emx2* antisense levels in cortico-cerebral neural precursors. An inverse correlation was found between levels of *Emx2OS* and *Emx2* transcripts, confirming that the former may normally contribute to down-regulation of the latter. Changes of *Emx2*-mRNA levels elicited by *Emx2OS*-RNAi are not reflected by the course of the corresponding pre-mRNA, that remains stable as found by intronic qRT-PCR. This further indicates that *Emx2OS*-dependent modulation of *Emx2* takes place post-transcriptionally. Antisense-promoted down-regulation of *Emx2* is weakened by Dicer1 knock-down, suggesting that Dicer1 might promote such process by “dicing” the *Emx2*-mRNA/*Emx2OS*-ncRNA hybrid, similarly to retrotransposon silencing [47], [49]. Consistently with this model, the concomitant up-regulation of *Emx2OS*-ncRNA caused by OS1-179(+) might reflect a reduced degradation rate of the endogenous antisense molecule, following the competition by OS1-179(+) for sense transcript binding.

Remarkably, *Emx2*OS-dependent down-regulation of *Emx2* is not limited to proliferating neural precursors. It takes place also in differentiating neuronal progenitors, where a surplus of *Emx2*OS-ncRNA is available to trigger this process (Fig. 5C, bottom left). Here, an up-regulation of key enzymes involved in sense RNA degradation, occurring during neuronal differentiation [66], might make the *Emx2*OS impact on *Emx2*-mRNA degradation predominant over its positive effects on sense transcription, so accelerating the shutting off of the sense transcript. Actually, the amplitude of *Emx2*OS-dependent *Emx2* downregulation is apparently not huge. Notwithstanding that, such regulation might be instrumental to proper progression of cortico-cerebral morphogenesis. In fact, as *Emx2* finely regulates the balance between symmetric, self-renewing divisions undergone by embryonic neural stem cells and asymmetrical, neuronogenic ones [12], an accurate regulation of its expression levels and, in particular, a finely tuned decay of its mRNA may be crucial to proper growth of the neocortical primordium [14]. A moderate contribution of *Emx2*OS-ncRNA to *Emx2*-mRNA destabilization might be just required, for optimal tuning of this process.

## Materials and Methods

### Ethics Statement

Animals handling and subsequent procedures were in accordance with European laws [European Communities Council Directive of November 24, 1986 (86/609/EEC)] and with National Institutes of Health guidelines.

### Animal Handling

Wt mice (strain CD1, purchased from Harlan-Italy) and *Emx2 null* mutants [**4**] used in this study are maintained at the SISSA-CBM mouse facility. Embryos were staged by timed breeding and vaginal plug inspection. Embryos (E10.5-E18.5) were harvested from pregnant dames killed by cervical dislocation.

### Preparation of Histological Samples

Dissected embryos were immersion-fixed overnight in 4% paraformaldehyde in 1X phosphate saline buffer (PBS) and then cryoprotected by immersion in 30% sucrose in PBS 1X overnight at 4°C. Tissues were frozen in OCT compound, sectioned at 10 µm in a cryostat, collected on slides SuperFrost Plus (Fischer), air dried for about 30 min, and stored at – 80°C until use.

### Cell Cultures

#### Primary cells

Cortical primordia and rhombo-spinal tracts were dissected from E12.5 mouse embryos and mechanically dissociated to single cells, by gentle pipetting. Dissociated neural precursor cells were cultured at 700 cells/µl, in DMEM/F12/Glutamax medium (Invitrogen™), integrated with N2 supplement (Invitrogen™), 1 mg/ml BSA, 0.6% w/v glucose, 2 µg/ml heparin, 10 pg/ml Fgf2, 20 pg/ml Egf, 1X Pen/Strept (Gibco), 10 pg/ml fungizone. Cultures were usually blocked and underwent RNA extraction 72 hours after dissection. When a longer culturing time was required, primary neurospheres were dissociated to single cells by trypsin-DNAseI and re-plated at the same initial density. When required, doxycycline, lithium chloride and cyclopamine were added to the culture medium, at 2 µg/µl, 1mM and 1 µM, respectively.

#### HeLa cells and NIH/3T3 cells

Cells were cultured in DMEM-Glutamax-I™ (Gibco) plus 10%FBS, according to standard protocols. When appropriate, α-Dicer (5′ GGCTT TTCAA TCATC CAGTG TTTCT 3′) and control (5′ TCTTT GTGAC CTACT AACTT TTCGG 3′) morpholinos were delivered to cells at 10 µM, by 6 µM EndoPorter™ carrier (GeneTools), according to manifacturer's instructions. When required, doxycycline was added to the culture medium, at 2 µg/µl.

### Immunofluorescence

Neural cells were detached form plates by 1X trypsin (Gibco) for 5 minutes at RT, centrifuged at 100 g for 7 min at RT, resuspended in Sato medium and finally left 60 min to attach to Superfrost® slides, previously kept for 60 min under 20 µg/ml poly-D-lysine at RT. Immunofluorescence was performed as previously described [66], with minor modifications. Briefly, after fixation by 4% paraformaldehyde for 10 min at 4°C and three washes in 1X PBS, 5 min each, cells were incubated for 1 h at RT under blocking mix (1X PBS; 10% FBS; 1 mg/ml BSA; 0.1% Triton-X100) and then incubated at 4°C overnight with primary antibody in blocking mix. Mouse anti-neuron-specific class III β-tubulin-primary antibody (clone Tuj1, Covance), was used, 1∶600. Immunoreactivity was revealed after a 2 h incubation with secondary Alexa-594 antibody, 1∶600.

### In Situ Hybridization

Not radioactive *in situ* hybridization was performed as previously described, with minor modifications [67]. Two riboprobes were used: *Emx2OS*, corresponding to mouse chromosome 19: nt 59,500,768-59,501,561, and *Reln*, corresponding to the 3.3 kb EcoRI fragment from the BS6 clone (a gift by A.Bulfone). Hybridized embryo sections were imaged and analyzed using a fluorescent Nikon (Tokyo, Japan) Eclipse 80i microscope and a DS-2MBWC digital microscope camera. All images were processed by Adobe Photoshop CS3 software.

### Quantitative RT-PCR

#### RNA preparation

RNA was extracted from CNS explants and cell cultures by Trizol™ (Invitrogen), according to manifacturer's instructions.

#### cDNA prepration

At least 1 µg of RNA from each sample was retrotranscribed by SuperScriptIII™ (Invitrogen) in the presence of random hexamers, according to manifacturer's instructions, with minor modifications. In the case of pre-mRNA levels evaluation, at least 4 µg of RNA preparation from each sample, previously treated by DNAseI™ (Promega), were used.

#### Quantitative PCR

1/25 of each cDNA sample was analyzed by the SybrGreen™ qPCR platform (Biorad). Each PCR reaction was run at least in triplicate and results averaged. Averages were further normalized against Tbp, except data in Fig. 5B (left and middle graphs), conversely normalized against GAPDH. Specifically in the case of pre-mRNA levels evaluation (reported in Fig. 5B, right graph), to reconcile sensitivity and specificity, 1/2 of each cDNA sample was linearly pre-amplified and 1/100 of the resulting primary reaction product was used as substrate of the subsequent quantitative PCR reaction, driven by nested, internal primers. Finally, supplementary amplifications on not-retrotranscribed samples were run, as negative controls.

#### Oligonucleotides

Genomic localization of oligonucleotides used in this study are shown in Fig. 1A. Amplicons and corresponding oligos were as follows: Emx2-mRNA (E2S/N2F: 5′ GGAAA GGAAG CAGCT GGCTC ACAGT CTCAG TCTTA C 3′; E2S/N2R: 5′ GTGGT GTGTC CCTTT TTTCT TCTGT TGAGA ATCTG AGCCT TC 3′); Emx2OS-ncRNA (Emx2OS/P1: 5′ CCCGC GCCCG GGTCA CTGAG ATGGC TTCG 3′; Emx2OS/P3: 5′ GATGA GCAGG TGAGT GGTAG ATGGT TGTAA GCTGT AC 3′); Emx2-pre-mRNA-primary PCR (I1: 5′ GTCTC TGAAG CTCGT TTGGG TTACT G 3′; I4: 5′ AGTGA GTGTA GAGCA GAGTT GAAGT CC 3′); Emx2-pre-mRNA-secondary PCR (I2: 5′ GCGAG GTCTT TGAAT CCTGT TTC 3′; I3: 5′ GCAGA GTTGA AGTCC AGTGA ACC 3′); Tbp-mRNA (Tbp-b/Fw: 5′ ATTCT CAAAC TCTGA CCACT GCACC GTTG 3′; Tbp-b/Rev: 5′ TTAGG TCAAG TTTAC AGCCA AGATT CACGG TAG 3′); Gapdh-mRNA (Gapd/FW: 5′ CAACA GCAAC TCCCA CTCTT CCACC TTCG 3′; Gapd/REV: 5′ GGTGG TCCAG GGTTT CTTAC TCCTT GGAGG 3′); Tbp-pre-mRNA-primary PCR (TBP-FW/EXT: 5′ CTCAG TTTGA TGGCT CAGTT TCC 3′; TBP-REV/EXT: 5′ GTATA ACCAG TTATT TATCC AGATC TC 3′); Tbp-pre-mRNA- secondary PCR (TBP-FW/INT: 5′ CAAAA GATGA AAACC CAGAA AACAG CC 3′; TBP-REV/INT: 5′ GTTTA CTGAA CGCTT GATTA TATAG 3′).

### Plasmids Construction

All basic DNA manipulations (extraction, purification, ligation) as well as bacterial cultures and transformation, media and buffer preparations were performed according to standard methods. DNAs were transformed in the E.Coli TOP-10 strains (Invitrogen).

### Loss-of-Function Constructs and Their Validation

To knock-down *Emx2*OS by RNAi, we selected the best two siRNAs suggested by the Invitrogen “BLOCK-iT™ RNAi Designer“ software (miR-a*Emx2*OS-542 and miR-a*Emx2*OS-774) plus the BLOCK-iT™ negative control-miR (miR-C) and compared their activities on Hela cells. For this purpose, we built up a set of three bicistronic plasmids, each harboring two distinct transcription units and designed to assay the activity of one specific miR. The former transcription unit, the “sensor”, included the CMV promoter, the DsRed2 coding sequence, the cDNA corresponding to the *Emx2*OS-ncRNA fragment targeted in silico by the two miRs above (Genbank AY117414.1: nt796-1289) and the SV40 polyA site. The latter unit, the “miR expressor”, harbored the CMV promoter, the eGFP coding sequence, a modified 230bp fragment from pri-mmu-miR-155 (where the miR-155 and miR-155* moieties are replaced by those of the artificial miR in order) and the TK polyA site. A fourth plasmid was built up as well, similar to the previous three, but harboring in *cis* sequences encoding for both miR-α*Emx2*OS-542 and miR-α*Emx2*OS-774 (Fig.1B). The pri-miR fragments included in the above mentioned plasmids were prepared as follows. Each of the two sequences 5′ TGCTG CATAT TTGCA CTTCT CCGAA GGTTT TGGCC ACTGA CTGAC CTTCG GAGGT GCAAA TATGC AGG 3′ and 5′ TGCTG CGAAC TTAGA CTCAG ATTCC CGTTT TGGCC ACTGA CTGAC GGGAA TCTGT CTAAG TTCGC AGG 3′ was cloned into pcDNA™6.2-GW/EmGFP-miR (Invitrogen), inbetween mmu-miR-155 flanking regions, according to manifacturer's instructions, and the resulting SalI-XhoI chimeric pri-miR cDNA fragments, encoding for miR-αEmx2OS-542 and miR-αEmx2OS-774, respectively, were obtained. Finally, the SalI-XhoI cDNA fragment from “pcDNA™6.2-GW/EmGFP-miR_neg_control_ plasmid” was used as control pri-miR.

We transfected HeLa cells with these four bicistronic plasmids, counted fluorescent cells and calculated for each plasmid the ratio between red cells and total fluorescent cells, as a comprehensive index of pri-miR processing efficiency and mature miR activity. The DsRed2^+^/(total fluorescent cells) ratio varied from 65% (negative control miR), to 39% (miR-α*Emx2OS*-542), 10% (miR-α*Emx2OS*-774) and 2% (miR-α*Emx2OS*-542 & miR-α*Emx2OS*-774), suggesting that the combination in *cis* of the two miRs, -542 and -774, would be the best choice (Fig. 5A). However, we noticed that, using the -542/-774 plasmid, the ratio between eGFP^+^ cells and total cells in the plate was consistently lower (data not shown), possibly because of enhanced Drosha-dependent destabilization of the chimaeric eGFP-cds/Pri-miR molecule. As this might pose serious problems in subsequent production of miR-encoding lentiviruses as well as in tracing cells infected by these virions, we prudently opted for simple miR-α*Emx2*OS-774 for the knocking-down experiments.

Finally, the pri-miR-α*Emx2*OS-774 and “control pri-miR” SalI/XhoI cDNA fragments were transferred into the SalI-digested, pCCLsin.PPT.hPGK.GFP.pre plasmid [68], inbetween the pPgk1/GFP and Wpre modules, so obtaining the genomic plasmids used for subsequent production of miR-expressor lentiviruses (Fig. 1B).

### Conditional Gain-of-Function Constructs

*Emx2*OS/*Emx2* RNA sequences were conditionally overexpressed by lentiviral vectors and TetON technology.

The “driver lentivector” encoded for a constitutively expressed rtTA transactivator. Its genomic plasmid was obtained by replacing the BamHI/SalI eGFP cassette of pCCLsin.PPT.hPGK.GFP.pre by a BamHI/XhoI rtTA2^S^-M2 module.

“Expressor viruses” encoded for a doxycycline-controlled chimaeric transgene, including the cDNA fragment subject of analysis plus an IRES/eGFP module. Its genomic plasmid was obtained starting from pCCLsin.PPT.hPGK.GFP.pre, replacing the pPgk1 promoter by an “tTA/rtTA responsive element, tight” (TREt), the eGFP cds by an IRES/eGFP module and finally cloning the cDNA in order inbetween TREt and IRES/eGFP. Two “expressor viruses” were built up: the former harbored the cDNA fragment corresponding to nucleotides 1–179 of *Emx2OS*-ncRNA [OS1-179(+)], the latter the reverse-complementary sequence [OS1-179(−)]. A third virus, not including any *Emx2* sense or antisense sequences was used as a negative control (Fig. 1C).

### Morpholino Reagents and Their Validation

Sequences of anti-Dicer1 morpholino and its reverse negative control were obtained from the Gene-Tools free design service (α-Dicer1 and C, respectively) and the former molecule was validated, by testing its capability to inhibit Dicer1-dependent pri-miR-124a maturation [56]. This was assayed by a DsRed2 sensor gene linked to a miR-124a responsive element, co-trasfected into NIH/3T3 cells with pri-miR-expressor plasmids (Fig. S1).

### Lentiviruses Preparation, Titration and Usage

Lentiviruses were prepared and titrated as previously described [68]–[70], with minor modifications.

Cell infections were performed without polybrene. In gain-of-function experiments, each virus was used at “multiplicity of infection” (m.o.i.) = 10, which resulted in trasduction of almost the totality of cortical precursors [71]. The m.o.i. was raised to 20 in loss-of-function experiments, in order to get a more robust downregulation of the target RNA.

### Statistical Analysis

All experiments were performed at least in biological triplicate. *Tbp*- (or *GAPDH*-) normalized qRT-PCR data relative to each treatment were further averaged and the corresponding s.e.m.'s ae determined. Resulting averages were finally normalized against the control treatment, as reported in Figure Legends. Statistical significance of differences among results was evaluated by one- or two-ways ANOVA.

## Supporting Information

Figure S1 Validation of α-Dicer1 morpholino in NIH/3T3 cells. (A) Rationale of the assay: α-Dicer1-dependent suppression of pri-miR-124a-dependent DsRed2 inhibition. (B) Molecular tools for validating α-Dicer1 activity: inserts of pri-miR-expressor and miR-124a-sensor plasmids; sequences of α-Dicer1 and control morpholinos. The miR-124a responsive element (miR-124a-RE) corresponds to the 477-bp 3′UTR fragment of mouse Lhx2-mRNA (chr2 (+):38224759-38225235); Pri-miR-124a corresponds to the 285-bp mouse Pri-miR-124(2) genomic fragment (chr3 (+):17695562-17695846); control Pri-miR contains the Pri-miR155 sequence from the BLOCK-iT™ expression vector (Invitrogen). (C) Rescue of miR124a-dependent DsRed2 inhibition by α-Dicer1 morpholino, in NIH/3T3 cells.
(8.78 MB TIF)
