# Supplementary figures and images for: Regulation of Emx2 Expression by Antisense Transcripts in Murine Cortico-Cerebral Precursors
Source: PLoS One. 2010 Jan 11;5(1):e8658. doi: 10.1371/journal.pone.0008658 (PMC2799550; doi:10.1371/journal.pone.0008658)

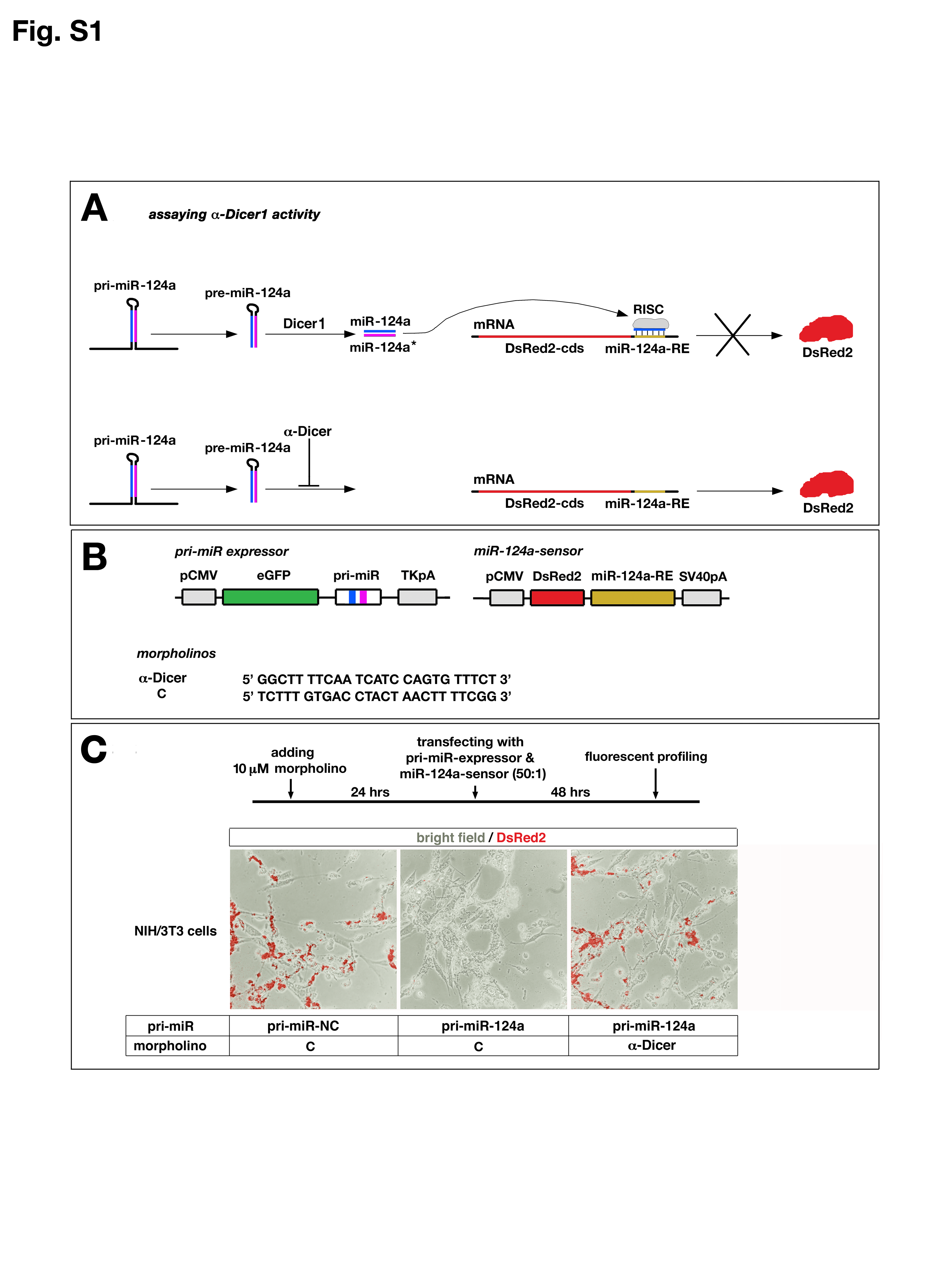

Supplement: Figure S1 — Validation of α-Dicer1 morpholino in NIH/3T3 cells. (A) Rationale of the assay: α-Dicer1-dependent suppression of pri-miR-124a-dependent DsRed2 inhibition. (B) Molecular tools for validating α-Dicer1 activity: inserts of pri-miR-expressor and miR-124a-sensor plasmids; sequences of α-Dicer1 and control morpholinos. The miR-124a responsive element (miR-124a-RE) corresponds to the 477-bp 3′UTR fragment of mouse Lhx2-mRNA (chr2 (+):38224759-38225235); Pri-miR-124a corresponds to the 285-bp mouse Pri-miR-124(2) genomic fragment (chr3 (+):17695562-17695846); control Pri-miR contains the Pri-miR155 sequence from the BLOCK-iT™ expression vector (Invitrogen). (C) Rescue of miR124a-dependent DsRed2 inhibition by α-Dicer1 morpholino, in NIH/3T3 cells. (8.78 MB TIF) [file pone.0008658.s001.tif]
